# Supplementary material for: Dynamics of Inflammatory and Neurodegenerative Biomarkers after Autologous Hematopoietic Stem Cell Transplantation in Multiple Sclerosis
Source: Int J Mol Sci. 2022 Sep 19;23(18):10946. doi: 10.3390/ijms231810946 (PMC9503241; doi:10.3390/ijms231810946)
Supplement: Supplementary file 1 [file ijms-23-10946-s001.zip › ijms-1852484-supplementary.pdf]

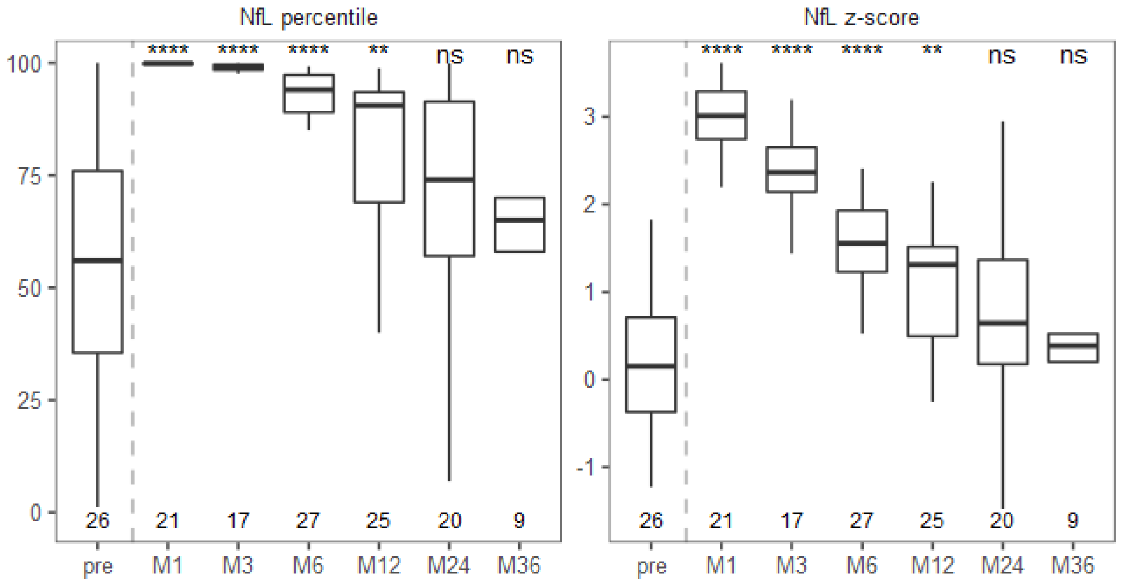

Supplementary Figure S1: NfL percentiles and z-scores show the same dynamics as absolute NfL values in the serum. Numbers at the bottom indicate number of samples included in respective boxplot. Global Kruskal-Wallis test was used first and in case of a significant difference, then a paired Wilcoxon signed-rank test as a post-hoc test with the pre-aHSCT time point as reference group. Significance levels were ** for $p \leq 0.01$, and **** for $p \leq 0.0001$.

Supplementary Table S1: Table showing patients and time points, cytokine level as well as CV value of each ELISA measurement.
CV: coefficient of variation; OD450: optical density at 450nm and AEB: average enzyme per bead.

	patient	timepoint	NfL [pg/ml]	AEB 1	AEB 2	CV
NfL in CSF	1	pre	421.9	0.35	0.35	1%
	1	M24	394.3	0.33	0.33	1%
	5	pre	360.0	0.30	0.30	1%
	5	M24	472.2	0.34	0.34	0%
	6	pre	378.6	0.31	0.32	1%
	6	M24	517.8	0.37	0.37	1%
	7	pre	266.0	0.23	0.22	3%
	7	M24	429.6	0.32	0.30	6%
	13	pre	374.7	0.32	0.30	5%
	13	M24	437.5	0.31	0.32	2%
	16	pre	543.0	0.45	0.45	1%
	16	M24	542.3	0.39	0.38	1%
	17	pre	7504.0	5.99	6.16	2%

	17	M24	339.2	0.25	0.24	4%
	18	pre	350.2	0.28	0.31	7%
	18	M24	424.5	0.30	0.31	1%
	19	pre	483.0	0.40	0.40	1%
	19	M24	462.6	0.33	0.34	3%
	21	pre	1847.6	1.49	1.52	1%
	21	M24	526.0	0.38	0.37	2%
	23	pre	836.9	0.68	0.69	1%
	23	M24	797.0	0.55	0.57	2%
NFL in serum	1	pre	7.9	0.08	0.07	8%
	1	M1	34.5	0.29	0.29	1%
	1	M6	10.3	0.09	0.09	0%
	1	M12	11.3	0.11	0.10	6%
	1	M24	8.0	0.08	0.07	4%
	2	M6	13.6	0.12	0.12	1%
	2	M12	10.5	0.10	0.09	9%
	2	M24	10.1	0.09	0.09	1%
	2	M36	7.4	0.06	0.07	5%
	3	pre	8.6	0.08	0.08	0%
	3	M6	15.1	0.13	0.13	1%
	3	M12	8.3	0.08	0.08	1%
	3	M24	9.7	0.08	0.08	6%
	3	M36	10.9	0.09	0.08	8%
	4	pre	6.2	0.06	0.06	7%
	4	M6	11.4	0.10	0.10	2%
	4	M12	10.8	0.10	0.09	9%
	4	M24	10.9	0.09	0.09	2%
	4	M36	7.5	0.06	0.06	0%
	5	pre	4.2	0.04	0.05	2%
	5	M3	16.7	0.15	0.14	3%
	5	M6	10.5	0.10	0.09	2%
	5	M12	9.6	0.09	0.08	5%
	5	M24	5.9	0.06	0.05	6%
	5	M36	6.0	0.05	0.06	3%
	6	pre	16.6	0.14	0.15	2%
	6	M6	22.3	0.18	0.20	6%
	6	M12	22.9	0.20	0.19	3%
	6	M24	25.2	0.19	0.18	5%
	6	M36	34.4	0.25	0.26	3%
	7	pre	7.0	0.06	0.07	7%
	7	M1	36.5	0.30	0.31	3%
	7	M6	10.8	0.09	0.09	1%
	7	M12	10.2	0.09	0.10	8%
	7	M24	13.2	0.11	0.10	2%
	7	M36	7.4	0.07	0.06	9%
	8	pre	5.9	0.06	0.06	2%
	8	M6	8.4	0.08	0.08	5%

8	M12	7.4	0.07	0.07	5%
9	pre	6.7	0.06	0.06	0%
9	M1	30.2	0.27	0.24	8%
9	M3	18.9	0.17	0.16	3%
9	M6	12.4	0.11	0.11	1%
9	M12	8.8	0.08	0.08	1%
9	M36	10.2	0.08	0.09	5%
10	pre	7.1	0.06	0.06	1%
10	M1	56.3	0.47	0.46	1%
10	M3	19.5	0.18	0.16	7%
10	M6	13.2	0.11	0.12	9%
10	M12	10.4	0.10	0.09	4%
10	M24	8.0	0.07	0.07	2%
10	M36	17.3	0.14	0.13	3%
11	pre	5.1	0.05	0.05	4%
11	M1	18.8	0.16	0.16	1%
11	M3	11.4	0.10	0.10	2%
11	M6	11.6	0.11	0.10	2%
11	M12	7.4	0.07	0.07	5%
11	M24	4.4	0.04	0.05	9%
11	M36	5.8	0.05	0.05	0%
12	pre	6.6	0.06	0.06	3%
12	M1	10.8	0.10	0.10	3%
12	M3	16.1	0.14	0.14	3%
12	M6	7.9	0.07	0.08	3%
12	M12	10.1	0.09	0.09	1%
13	pre	8.2	0.08	0.07	6%
13	M1	18.0	0.15	0.16	2%
13	M3	16.0	0.14	0.14	1%
13	M6	11.5	0.10	0.10	0%
13	M12	11.7	0.11	0.10	11%
13	M24	7.8	0.07	0.07	1%
14	pre	3.9	0.04	0.04	7%
14	M1	8.3	0.08	0.07	10%
14	M3	24.4	0.21	0.20	3%
14	M6	8.5	0.08	0.08	5%
14	M12	5.3	0.05	0.05	4%
14	M24	4.8	0.04	0.05	4%
15	pre	8.6	0.08	0.08	0%
15	M1	122.5	1.00	1.00	0%
15	M3	21.8	0.18	0.19	4%
15	M6	15.7	0.13	0.14	3%
15	M12	13.8	0.10	0.11	4%
16	pre	8.9	0.08	0.09	6%
16	M1	68.2	0.56	0.57	1%
16	M3	18.3	0.16	0.16	1%
16	M6	13.2	0.12	0.11	4%
16	M12	9.0	0.08	0.08	2%

16	M24	22.8	0.17	0.17	2%
17	pre	78.7	0.68	0.61	7%
17	M1	78.9	0.57	0.58	0%
17	M3	42.3	0.35	0.36	2%
17	M6	17.7	0.15	0.16	3%
17	M12	15.5	0.11	0.13	7%
17	M24	9.3	0.07	0.08	8%
18	pre	7.6	0.07	0.07	1%
18	M1	39.7	0.34	0.32	3%
18	M3	23.4	0.20	0.20	1%
18	M6	15.5	0.13	0.14	7%
18	M12	13.0	0.10	0.10	2%
18	M24	9.1	0.08	0.08	0%
19	pre	9.9	0.09	0.09	5%
19	M1	37.1	0.31	0.31	0%
19	M6	14.6	0.13	0.13	3%
19	M12	12.0	0.09	0.10	5%
19	M3	18.7	0.14	0.14	2%
19	M24	8.6	0.07	0.08	7%
20	pre	12.0	0.11	0.11	0%
20	M1	27.4	0.23	0.24	3%
20	M3	18.3	0.16	0.16	0%
20	M6	14.1	0.12	0.13	4%
20	M12	10.5	0.09	0.08	2%
20	M24	7.3	0.07	0.06	5%
21	pre	18.5	0.16	0.16	1%
21	M1	19.4	0.16	0.17	4%
21	M6	13.5	0.11	0.10	2%
21	M12	10.4	0.08	0.09	3%
21	M24	7.9	0.07	0.07	1%
22	pre	9.6	0.09	0.09	4%
22	M1	37.3	0.32	0.30	3%
22	M6	14.3	0.11	0.11	3%
22	M24	11.1	0.09	0.09	0%
23	pre	7.6	0.07	0.07	3%
23	M1	55.5	0.47	0.44	4%
23	M3	24.0	0.21	0.19	7%
23	M6	12.7	0.10	0.10	0%
23	M12	10.3	0.09	0.08	4%
23	M24	12.5	0.10	0.10	2%
24	pre	7.2	0.07	0.07	5%
24	M1	38.7	0.32	0.32	1%
24	M3	20.1	0.17	0.17	1%
24	M6	11.6	0.10	0.09	9%
24	M12	9.6	0.08	0.08	5%
25	pre	4.8	0.05	0.05	8%
25	M1	52.2	0.42	0.44	4%
25	M3	25.9	0.22	0.22	2%

	patient	timepoint	GFAP [pg/ml]	AEB 1	AEB 2	CV
	25	M6	14.1	0.11	0.11	2%
	25	M12	8.8	0.07	0.08	9%
	26	pre	6.7	0.06	0.06	2%
	26	M1	32.0	0.27	0.27	1%
	26	M3	21.6	0.16	0.16	2%
	26	M6	11.0	0.09	0.09	2%
	26	M12	8.5	0.07	0.07	2%
	26	M24	9.4	0.08	0.08	1%
	27	pre	6.9	0.06	0.07	5%
	27	M1	45.3	0.33	0.33	0%
	27	M6	11.2	0.09	0.09	2%

	patient	timepoint	GFAP [pg/ml]	AEB 1	AEB 2	CV
GFAP in CSF	1	pre	1091.0	0.54	0.56	1%
	1	M24	1326.8	0.67	0.66	1%
	5	pre	628.7	0.32	0.34	4%
	5	M24	7968.8	0.34	0.35	2%
	6	pre	757.5	0.37	0.41	7%
	6	M24	10667.7	0.45	0.45	1%
	7	pre	721.7	0.36	0.38	4%
	7	M24	5769.9	0.26	0.25	3%
	13	pre	1340.9	0.68	0.66	2%
	13	M24	15551.8	0.63	0.65	2%
	16	pre	1127.6	0.56	0.58	2%
	16	M24	12922.0	0.55	0.53	3%
	17	pre	1375.6	0.67	0.70	3%
	17	M24	6876.0	0.30	0.29	2%
	18	pre	1210.9	0.58	0.63	5%
	18	M24	11960.4	0.47	0.53	8%
	19	pre	1090.2	0.54	0.56	2%
	19	M24	13851.3	0.59	0.56	3%
	21	pre	750.0	0.39	0.38	0%
	21	M24	5306.4	0.24	0.23	1%
	23	pre	1018.3	0.51	0.52	1%
	23	M24	9417.3	0.40	0.40	0%
GFAP in serum	1	pre	92.5	0.06	0.06	2%
	1	M1	320.3	0.17	0.18	4%
	1	M6	109.1	0.07	0.07	0%
	1	M12	178.8	0.10	0.10	1%
	1	M24	119.5	0.07	0.07	5%
	2	M6	124.4	0.07	0.07	2%
	2	M12	130.8	0.08	0.08	1%
	2	M24	132.1	0.08	0.08	1%
	2	M36	117.1	0.06	0.06	0%
	3	pre	102.7	0.06	0.07	8%
	3	M6	139.2	0.08	0.08	5%

3	M12	85.9	0.05	0.05	3%
3	M24	124.0	0.06	0.06	1%
3	M36	124.0	0.07	0.06	3%
4	pre	87.0	0.05	0.06	5%
4	M6	110.2	0.06	0.07	10%
4	M12	112.1	0.07	0.07	1%
4	M24	129.0	0.07	0.06	5%
4	M36	101.5	0.06	0.05	4%
5	pre	137.5	0.08	0.08	0%
5	M3	132.3	0.07	0.08	5%
5	M6	108.5	0.07	0.06	2%
5	M12	119.5	0.07	0.07	7%
5	M24	88.2	0.05	0.05	2%
5	M36	109.3	0.05	0.06	8%
6	pre	171.8	0.10	0.10	4%
6	M6	186.7	0.10	0.11	12%
6	M12	193.5	0.11	0.11	3%
6	M24	206.9	0.10	0.10	1%
6	M36	257.5	0.12	0.12	1%
7	pre	77.0	0.05	0.05	3%
7	M1	192.4	0.10	0.12	12%
7	M6	96.2	0.05	0.05	1%
7	M12	97.0	0.06	0.06	1%
7	M24	65.1	0.04	0.04	2%
7	M36	113.3	0.06	0.06	5%
8	pre	30.0	0.02	0.02	4%
8	M6	45.1	0.03	0.03	4%
8	M12	44.1	0.03	0.03	2%
9	pre	75.8	0.04	0.05	15%
9	M1	155.6	0.09	0.09	6%
9	M3	108.1	0.07	0.06	8%
9	M6	94.7	0.06	0.06	6%
9	M12	76.3	0.05	0.05	6%
9	M36	118.4	0.06	0.06	1%
10	pre	38.6	0.02	0.03	9%
10	M1	210.1	0.11	0.13	13%
10	M3	61.3	0.04	0.04	0%
10	M6	50.1	0.03	0.04	8%
10	M12	61.0	0.04	0.04	3%
10	M24	60.6	0.03	0.04	9%
10	M36	55.6	0.04	0.03	13%
11	pre	38.8	0.03	0.03	10%
11	M1	126.6	0.08	0.07	6%
11	M3	55.7	0.04	0.04	3%
11	M6	74.7	0.05	0.05	5%
11	M12	240.4	0.13	0.14	8%
11	M24	60.0	0.02	0.04	25%
11	M36	61.3	0.03	0.04	10%

12	pre	70.6	0.04	0.05	5%
12	M1	53.8	0.03	0.04	9%
12	M3	82.5	0.05	0.05	0%
12	M6	72.2	0.04	0.05	10%
12	M12	62.4	0.04	0.04	5%
13	pre	121.6	0.07	0.07	4%
13	M1	97.5	0.06	0.06	1%
13	M3	119.6	0.07	0.08	8%
13	M6	103.9	0.06	0.06	2%
13	M12	133.2	0.08	0.07	8%
13	M24	111.1	0.06	0.06	5%
14	pre	62.6	0.04	0.04	4%
14	M1	81.7	0.05	0.05	4%
14	M3	144.7	0.08	0.09	2%
14	M6	79.2	0.05	0.05	2%
14	M12	60.2	0.04	0.04	2%
14	M24	55.5	0.03	0.03	2%
15	pre	71.0	0.05	0.05	2%
15	M1	130.4	0.08	0.08	0%
15	M3	78.4	0.05	0.05	8%
15	M6	99.2	0.06	0.06	6%
15	M12	82.2	0.04	0.05	3%
16	pre	76.5	0.05	0.05	2%
16	M1	328.2	0.19	0.17	9%
16	M3	97.6	0.06	0.06	3%
16	M6	98.9	0.06	0.06	1%
16	M12	81.9	0.05	0.05	0%
16	M24	82.2	0.05	0.04	5%
17	pre	145.5	0.09	0.08	4%
17	M1	430.1	0.19	0.19	2%
17	M3	179.2	0.10	0.10	3%
17	M6	180.1	0.10	0.10	1%
17	M12	188.0	0.09	0.09	0%
17	M24	116.7	0.06	0.06	5%
18	pre	58.7	0.04	0.04	3%
18	M1	141.2	0.08	0.08	2%
18	M3	75.9	0.05	0.05	6%
18	M6	101.8	0.06	0.06	2%
18	M12	88.7	0.05	0.05	6%
18	M24	84.0	0.05	0.05	1%
19	pre	83.2	0.05	0.05	1%
19	M1	186.3	0.11	0.10	5%
19	M6	114.1	0.07	0.07	4%
19	M12	118.3	0.06	0.06	2%
19	M3	129.2	0.07	0.07	1%
19	M24	80.5	0.05	0.04	15%
20	pre	57.4	0.04	0.04	1%
20	M1	135.5	0.08	0.08	3%

20	M3	82.8	0.05	0.05	1%
20	M6	102.1	0.06	0.07	7%
20	M12	81.0	0.04	0.05	2%
20	M24	56.0	0.03	0.04	7%
21	pre	81.9	0.05	0.05	3%
21	M1	130.9	0.08	0.08	1%
21	M6	69.1	0.04	0.04	4%
21	M12	72.5	0.04	0.04	2%
21	M24	92.1	0.05	0.05	8%
22	pre	163.3	0.09	0.09	1%
22	M1	281.9	0.16	0.15	3%
22	M6	211.2	0.10	0.10	2%
22	M24	145.0	0.07	0.08	7%
23	pre	56.6	0.04	0.04	4%
23	M1	197.0	0.12	0.11	7%
23	M3	83.4	0.05	0.05	1%
23	M6	58.5	0.03	0.04	6%
23	M12	73.2	0.04	0.04	1%
23	M24	101.4	0.05	0.06	7%
24	pre	74.0	0.04	0.05	9%
24	M1	226.2	0.12	0.13	7%
24	M3	95.3	0.06	0.06	2%
24	M6	110.4	0.06	0.05	8%
24	M12	152.6	0.08	0.07	3%
25	pre	86.1	0.05	0.05	2%
25	M1	361.3	0.18	0.21	9%
25	M3	155.8	0.09	0.09	4%
25	M6	136.6	0.07	0.07	6%
25	M12	104.5	0.05	0.06	2%
26	pre	127.6	0.07	0.08	5%
26	M1	180.9	0.09	0.11	13%
26	M3	166.2	0.08	0.08	2%
26	M6	111.9	0.06	0.06	6%
26	M12	128.8	0.07	0.06	2%
26	M24	147.0	0.08	0.07	7%
27	pre	92.5	0.06	0.06	5%
27	M1	206.1	0.10	0.10	4%
27	M6	81.4	0.04	0.05	5%

	patient	timepoint	CHI3L1 [ng/ml]	well 1 OD450	well 2 OD450	CV
CHI3L1 in CSF	1	pre	365.5	2.5244	2.651	3%
	1	M24	140.0	1.12	1.26	8%
	5	pre	233.6	1.74	2.14	15%
	5	M24	203.6	1.48	1.60	6%
	6	pre	534.1	3.48	3.83	7%
	6	M24	378.8	2.67	3.29	15%
	7	pre	120.4	0.99	1.10	7%

	7	M24	163.1	1.27	1.49	11%
	13	pre	165.4	1.31	1.27	2%
	13	M24	182.6	1.46	1.34	6%
CHI3L1 in serum	1	pre	36.9	0.39	0.43	7%
	1	M6	28.0	0.35	0.35	1%
	1	M12	24.7	0.32	0.36	8%
	1	M24	33.2	0.37	0.40	7%
	5	pre	29.0	0.36	0.42	12%
	5	M6	27.3	0.33	0.35	4%
	5	M12	25.6	0.32	0.35	6%
	5	M24	31.8	0.38	0.46	14%
	6	pre	88.7	0.86	0.77	7%
	6	M6	62.0	0.62	0.56	6%
	6	M12	129.7	1.05	1.21	10%
	6	M24	85.3	0.75	1.02	22%
	7	pre	0.0	0.25	0.28	9%
	7	M6	21.9	0.31	0.30	1%
	7	M12	0.0	0.28	0.25	8%
	7	M24	31.8	0.38	0.44	11%
	13	pre	28.3	0.35	0.36	2%
	13	M6	0.0	0.24	0.25	3%
	13	M12	20.1	0.30	0.28	7%
	13	M24	0.0	0.26	0.30	11%
	patient	timepoint	CXCL10 [pg/ml]	well 1 OD450	well 2 OD450	CV
CXCL10 in CSF	1	Pre	16.8	0.16	0.12	19%
	1	M24	12.8	0.12	0.10	18%
	5	Pre	18.3	0.20	0.13	31%
	5	M24	35.0	0.36	0.24	28%
	6	Pre	30.2	0.31	0.21	28%
	6	M24	112.6	0.99	0.78	17%
	7	Pre	14.3	0.14	0.11	19%
	7	M24	36.2	0.33	0.25	21%
	13	Pre	19.2	0.16	0.14	12%
	13	M24	45.6	0.42	0.31	21%
	16	Pre	55.2	0.45	0.38	12%
	16	M24	52.9	0.35	0.37	5%
	17	Pre	22.2	0.15	0.18	9%
	17	M24	22.0	0.20	0.15	17%
	18	Pre	187.2	1.26	1.33	3%
	18	M24	270.7	1.92	1.99	3%
	19	Pre	24.0	0.20	0.17	12%
	19	M24	25.3	0.32	0.18	41%
	1	Pre	231.2	1.67	1.63	2%

	1	M24	218.7	1.53	1.58	2%
	5	Pre	95.8	0.66	0.76	10%
	5	M3	143.5	1.16	1.00	10%
	5	M24	131.2	0.92	0.91	1%
	6	Pre	53.0	0.36	0.43	13%
	6	M24	117.2	0.82	0.80	2%
	7	Pre	52.4	0.35	0.36	3%
	7	M1	296.4	2.11	2.18	3%
	7	M24	127.5	0.89	1.07	13%
	9	Pre	79.5	0.50	0.51	2%
	9	M1	160.7	1.08	1.10	1%
	9	M12	143.2	0.96	0.97	0%
	10	Pre	98.7	0.64	0.63	1%
	10	M1	269.0	1.88	2.00	4%
	10	M3	112.9	0.71	0.77	6%
	10	M24	61.0	0.40	0.36	7%
	11	Pre	127.8	0.83	0.87	3%
	11	M1	331.3	2.39	2.51	3%
	11	M3	220.0	1.54	1.56	1%
	11	M24	126.7	0.85	0.83	2%
	13	Pre	50.7	0.51	0.34	27%
	13	M1	798.5	1.15	1.07	5%
	13	M24	74.6	0.61	0.51	12%
CXCL10 in serum	14	Pre	58.7	0.36	0.36	0%
	14	M1	264.7	1.91	1.91	0%
	14	M24	144.8	0.96	0.99	2%
	16	Pre	61.0	0.43	0.40	6%
	16	M1	150.5	1.02	1.08	4%
	16	M3	94.3	0.65	0.65	0%
	16	M24	60.1	0.41	0.69	36%
	17	Pre	297.9	2.10	2.21	3%
	17	M1	1744.0	2.39	2.42	1%
	17	M24	192.6	1.57	1.36	10%
	18	Pre	77.6	0.57	0.49	10%
	18	M1	1749.5	2.36	2.46	3%
	18	M24	129.2	0.86	0.85	1%
	19	Pre	67.9	0.46	0.47	2%
	19	M1	97.2	0.65	0.69	4%
	19	M24	40.5	0.27	0.28	3%
	20	Pre	110.6	0.72	0.73	0%
	20	M1	195.6	1.36	1.36	0%
	20	M24	187.6	1.29	1.31	1%
	21	Pre	53.1	0.31	0.34	6%
	21	M1	133.3	0.86	0.92	5%
	21	M12	60.7	0.36	0.39	5%
	22	Pre	162.5	1.08	1.13	3%
	22	M1	264.7	1.83	1.98	6%
	22	M24	104.5	0.67	0.69	3%

23	Pre	66.8	0.40	0.44	6%
23	M1	129.1	0.83	0.89	5%
23	M12	72.5	0.46	0.51	8%
24	Pre	139.1	0.93	1.04	7%
24	M1	236.3	1.65	1.71	3%
24	M12	84.8	0.55	0.54	1%
25	Pre	90.9	0.58	0.59	1%
25	M1	188.0	1.25	1.35	5%
25	M12	98.9	0.63	0.65	3%
26	Pre	94.0	0.58	0.63	5%
26	M1	179.2	1.24	1.23	1%
26	M12	107.5	0.70	0.78	7%
27	pre	83.2	0.50	0.47	3%
27	M1	517.2	3.14	3.11	1%
27	M6	146.4	0.87	0.85	2%
28	pre	77.1	0.46	0.44	3%
28	M1	168.5	1.00	0.98	2%
28	M3	69.0	0.40	0.40	1%
28	M6	154.9	0.89	0.93	3%
28	M12	127.3	0.75	0.74	1%
31	pre	122.2	0.72	0.71	1%
31	M1	386.8	2.31	2.32	0%
31	M3	261.7	1.56	1.54	1%
31	M6	219.7	1.31	1.29	1%
31	M12	177.1	1.04	1.04	0%
32	pre	87.6	0.53	0.49	6%
32	M1	949.1	1.34	1.29	3%
32	M3	278.9	1.70	1.61	4%
32	M6	146.9	0.87	0.86	1%
32	M12	208.8	1.25	1.22	2%
34	pre	126.3	0.76	0.72	4%
34	M1	270.9	1.63	1.59	2%
34	M6	152.9	0.90	0.90	0%
36	pre	102.6	0.59	0.60	1%
36	M1	133.7	0.79	0.78	1%
36	M3	148.5	0.87	0.87	1%
36	M6	296.3	1.75	1.77	#NAME?

	patient	timepoint	CXCL9 [pg/ml]	well 1 OD450	well 2 OD450	CV
CXCL9 in CSF	1	Pre	7.6	0.03	0.03	0%
	1	M24	14.0	0.03	0.03	1%
	5	Pre	25.0	0.05	0.05	0%
	5	M24	27.1	0.05	0.05	2%
	6	Pre	0.0	0.02	0.02	16%
	6	M24	7.1	0.03	0.02	7%
	7	Pre	0.0	0.02	0.02	1%
	7	M24	5.3	0.03	0.02	13%

	13	Pre	0.0	0.02	0.02	3%
	13	M24	7.1	0.03	0.03	10%
	16	Pre	11.0	0.03	0.03	6%
	16	M24	8.0	0.03	0.03	11%
	17	Pre	6.0	0.02	0.02	0%
	17	M24	0.0	0.02	0.02	2%
	19	Pre	0.0	0.03	0.02	15%
	19	M24	5.9	0.05	0.02	47%
CXCL9 in serum	1	Pre	76.9	0.11	0.14	17%
	1	M1	964.8	1.37	1.35	1%
	1	M24	75.3	0.11	0.18	34%
	5	Pre	76.6	0.11	0.11	4%
	5	M3	202.6	0.27	0.28	3%
	5	M24	223.1	0.31	0.30	2%
	6	Pre	94.9	0.13	0.14	2%
	6	M24	207.9	0.28	0.50	39%
	7	Pre	101.8	0.14	0.19	20%
	7	M1	116.4	0.16	0.20	16%
	7	M24	190.9	0.25	0.27	4%
	13	Pre	28.2	0.05	0.05	3%
	13	M1	235.6	0.32	0.36	8%
	13	M24	53.7	0.08	0.08	4%
	16	Pre	41.4	0.06	0.07	4%
	16	M1	93.1	0.13	0.16	15%
	16	M3	144.0	0.20	0.20	1%
	16	M24	143.4	0.21	0.19	6%
	17	Pre	116.8	0.22	0.16	20%
	17	M1	244.2	0.49	0.33	26%
	17	M24	185.1	0.35	0.25	23%
	19	Pre	34.0	0.06	0.06	8%
	19	M1	24.0	0.04	0.05	1%
	19	M24	109.2	0.15	0.16	3%

	patient	timepoint	CXCL13 [pg/ml]	well 1 OD450	well 2 OD450	CV
CXCL13 in CSF	1	Pre	0.0	0.03	0.04	12%
	1	M24	0.0	0.03	0.03	6%
	5	Pre	0.0	0.03	0.03	12%
	5	M24	0.0	0.02	0.02	3%
	6	Pre	0.0	0.02	0.03	7%
	6	M24	0.0	0.02	0.03	30%
	7	Pre	0.0	0.02	0.03	31%
	7	M24	0.0	0.02	0.02	15%
	13	Pre	0.0	0.03	0.03	3%
	13	M24	0.0	0.02	0.02	8%
	16	Pre	0.0	0.02	0.01	15%
	16	M24	0.0	0.02	0.02	6%

	17	Pre	0.0	0.01	0.02	34%
	17	M24	0.0	0.02	0.02	15%
	19	Pre	0.0	0.02	0.02	14%
	19	M24	0.0	0.03	0.02	21%
CXCL13 in serum	1	Pre	211.1	1.27	1.30	2%
	1	M1	57.3	0.38	0.43	9%
	1	M24	37.7	0.26	0.26	1%
	5	Pre	22.0	0.16	0.17	3%
	5	M3	58.6	0.40	0.37	4%
	5	M24	45.3	0.31	0.30	1%
	6	Pre	31.4	0.22	0.26	12%
	6	M24	54.2	0.41	0.36	10%
	7	Pre	52.4	0.35	0.39	8%
	7	M1	25.4	0.18	0.19	4%
	7	M24	25.8	0.19	0.25	20%
	13	Pre	12.2	0.10	0.12	12%
	13	M1	27.7	0.20	0.25	16%
	13	M24	46.5	0.31	0.32	2%
	16	Pre	31.4	0.22	0.22	0%
	16	M1	44.5	0.31	0.30	2%
	16	M3	49.2	0.34	0.32	5%
	16	M24	50.9	0.34	0.41	13%
	17	Pre	39.3	0.26	0.27	2%
	17	M1	74.5	0.48	0.54	9%
	17	M24	52.1	0.34	0.35	2%
	19	Pre	26.2	0.19	0.19	1%
	19	M1	25.4	0.19	0.22	12%
	19	M24	50.8	0.34	0.38	8%
